# Resilience of Self-Organised and Top-Down Planned Cities—A Case Study on London and Beijing Street Networks

**DOI:** 10.1371/journal.pone.0141736

**Published:** 2015-12-18

**Authors:** Jiaqiu Wang

**Affiliations:** Centre for Advanced Spatial Analysis(CASA), University College London, London, United Kingdom; Beihang University, CHINA

## Abstract

The success or failure of the street network depends on its reliability. In this article, using resilience analysis, the author studies how the shape and appearance of street networks in self-organised and top-down planned cities influences urban transport. Considering London and Beijing as proxies for self-organised and top-down planned cities, the structural properties of London and Beijing networks first are investigated based on their primal and dual representations of planar graphs. The robustness of street networks then is evaluated in primal space and dual space by deactivating road links under random and intentional attack scenarios. The results show that the reliability of London street network differs from that of Beijing, which seems to rely more on its architecture and connectivity. It is found that top-down planned Beijing with its higher average degree in the dual space and assortativity in the primal space is more robust than self-organised London using the measures of maximum and second largest cluster size and network efficiency. The article offers an insight, from a network perspective, into the reliability of street patterns in self-organised and top-down planned city systems.

## Introduction

Cities, as complex systems, exhibit a wide diversity both in their overall shape (circular, sprawling, linear, or even fractal) and in the appearance of their street networks (regular, treelike, and organic) [1]. Such diversity of urban morphologies is closely linked to the urbanisation process. Recent studies in the evolution of cities through street networks show this urbanisation is either a process of densification governed by self-organisation (i.e. out of the control of any central agencies) or a top-down planned process [2–5]. Recently, the robustness of cities has become a new focus for thinking about both the short and long-term futures of city systems [6]. One of the aspects in urban studies is to consider cities as networks [7]. Cities are network world including tube network, street network, gas network, power network, telephone network, social network, and so on. In the last decade the study of network robustness has become an important area of research in many disciplines such as biology [8], computer science [9], physics [10–15], urban science [6, 7], and so on. Street networks, as a backbone of cities, have always been an important research object in understanding evolution of cities [4, 15–19]. No matter what kind of networks in cities, it is crucial to understand how networks perform and when networks collapse in the face of disruption. The process to understand the robustness of networked systems is closely relevant to the study of percolation on networks, which has been studied in the literatures [10–12, 14, 15]. By far the majority of work has focused on the percolation threshold of networked systems in primal space. In the study of street networks, the primal space is a graph representation of the networks where intersections are vertices and segments are edges. However, previous works have shown dual space is a more suitable representation of street networks, which is employed in consideration of describing complexity of urban street networks [2, 18–21]. Here the dual space is a graph representation of street networks where roads are vertices and intersections are edges.

Inspired by the significant number of works done on the network resilience and the recent development of the robustness of cities, the research is going to understand the resilience of self-organised and top-down planned city systems from a network perspective. The aim of the article is to study how the shape and appearance of urban street networks in self-organised and top-down planned city systems impacts on urban transport by resilience analysis in the primal space and dual space. In this study, London and Beijing are chosen as proxies [21, 22] for self-organised and top-down planned cities respectively by studying the robustness of tree-like and grid-like street networks. The topological and geometrical properties of London and Beijing networks first are investigated to see how to differentiate between London and Beijing. Graph theory offers a natural way to study the properties of street networks. A graph by *G* ≡ {*V*,*E*} is a set of vertices *V* and a set of edges *E* representing the relations between the pairs of vertices. Urban transport systems as networks can be represented as *planar* graphs and these have been widely studied [21, 23, 24]. There are two kinds of graph representations that are primal and dual. The primal graph is a straightforward representation of street network where intersections are vertices and street segments are edges. It is well accepted that such a representation is not sufficient to describe the complexity of street networks although it retains the geometric patterns and geographical properties (i.e geographical positions, street lengths and so on). However, such complexity of street network can be represented in the dual space [19–21, 25]. In the dual representation of street network, successive street segments are identified as constitutive transportation unit (i.e. road). In the dual graph, a road is a vertex and intersection is edge. Such representation gives prominence to topological relationship of transportation entities by ignoring geometrical properties of roads. By analysing the properties of street network in the both primal and dual space, one can investigate the complexity of street network in both topological and geometrical space. Then, the reliability of the London and Beijing networks is analysed by simulating random failure and intended attack following the method introduced by [26]. Starting from a primal graph with its associated dual graph, an edge is erased in the primal space and then obtain a new primal and dual graph. In this process, relevant quantities (e.g. maximum and second largest cluster size, network efficiency, diameter, average degree, etc) are monitored to see how they vary in the primal and dual space.

The history of London dates back over 2000 years, to the Roman invasion of AD 43. However, Beijing has a history of more than 3,000 years, and has functioned as capital for more than 850 years. Although London and Beijing have both been planned since being established, the two cities experienced different historical development (or urbanisation) processes. For instance, the development of London suffered historically from damage caused by fire, and aerial bombardment. After the Great Fire there was no great network or plot redesign but after World War II although there was some localised plot redesign in the Comprehensive Development Areas (CDAs) and there was new road construction of the M25, M4, Westway etc., the basic radial structure of existing main routes remained intact. The historical development of London can therefore be understood as resulting from a process of self-organisation [18]. Investigation into the street network of London shows that its topological structure tends to be self-organised, varying between a growing random city and a grid-like city [21]. By contrast, in the development process of Beijing, the original inner city (including the Forbidden City), and its peripheral areas survived several periods of chaos caused by civil war during the dynastic struggles in Chinese history and have not changed much since their creation. In spite of this, in recent decades the two capital cities have grown rapidly, particularly in Beijing. Nevertheless, the backbone of the street networks of each city still remain its original forms [18, 27]. Research shows that as the city evolves, its street network is preserved, frozen in time. In particular, some street networks maintain their overall geometry for hundreds of years and are thus extremely resilient to change [3, 17, 28].

**Fig 1:**
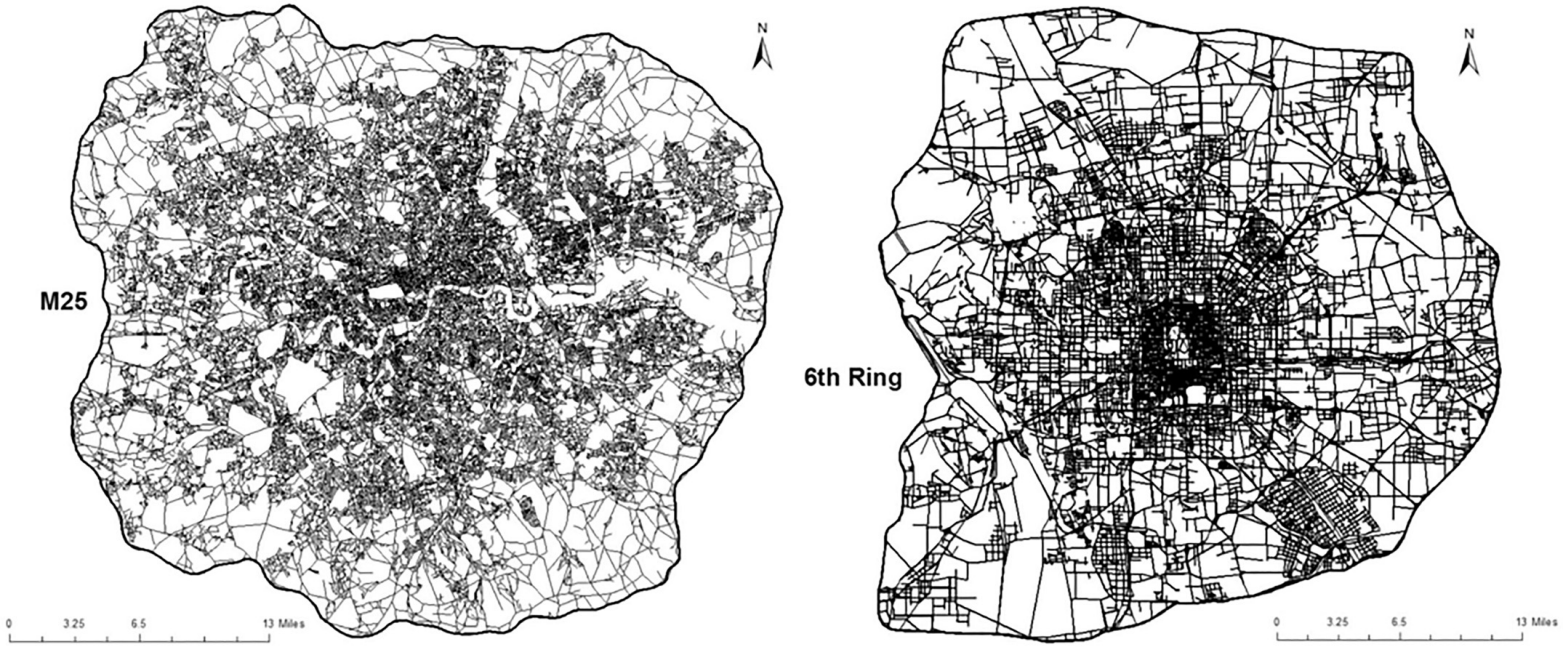
Street networks of metropolitan area delimited by orbital roads— M25 for London and 6th ring for Beijing. Left panel: London street network (source: UK Ordance Survey (see S1 Appendix), Using: Meridian 2 Digimap Ordnance Survey Service). London street network is governed by dendritic pattern. Right panel: Beijing street network (source: Open Street Map (OSM) (see S1 Appendix), Using: Road Shapefile Layer). Beijing street network is dominated by grid-like pattern.

In Fig 1, the distinct patterns of the London (a) and Beijing (b) street networks are displayed in their metropolitan areas bounded by the M25 orbital road in London and the 6^th^ ring in Beijing. There are two basic structures which can be observed in planar transportation networks: tree-like networks and grid-like networks [29, 30]. From Fig 1, it appears that the Beijing street network is dominated by a grid-like structure whereas the London street network tends to a more dendritic and less grid-like structure. This becomes clear when their topology and geometry are characterised based on their primal and dual representations.

## Analysis

Primal graphs are created for each city and the properties first are characterised by measures in the primal space. *’Connectivity’* is a measure of linkage of street network by calculating ratio (*E*/*V*) of the number of edges (*E*) to the number of vertices (*V*) in a planar graph [30]. In the case of London, *V* is 74557 and *E* is 107194 while in the case of Beijing, *V* is 44770 and *E* is 67941. Accordingly, value of *’connectivity’* equals to 1.518 for Beijing, which is higher than the value of 1.438 for London. This means that Beijing has greater linkage when compared to London. For the structures of street networks, previous studies show they can be identified and evaluated by the measures of *gridness*, *treeness*, *ringness*, *webness*,*Shannon entropy* [17, 23, 29–31],typology [32]. That is, a well planned city is likely to have a square-like or grid-like structure in contrast to a self-organised city’s dendritic structure. To quantify differences between self-organised and top-down planned cities, *’meshness’* and ‘organic’ indicators are utilised to measure forms of cities.

### Meshness and Organic

Meshness of a planar graph is defined as a ratio of the existing and the maximum number of faces in a graph [23, 33]:

$$M=\frac{F}{2V-5}$$ (1)

**Fig 2:**
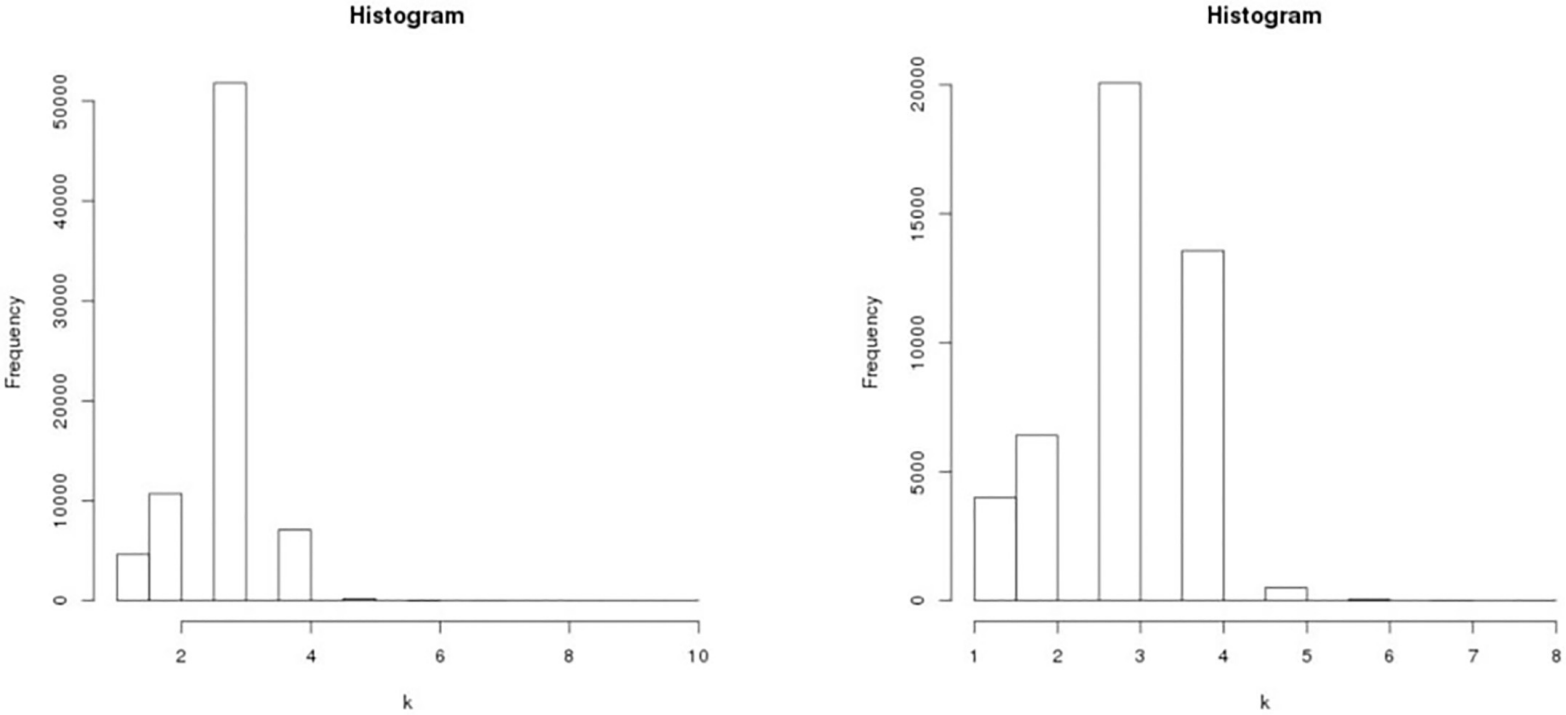
Histogram of degree distribution in the primal space for London (Left) and Beijing (Right).

F=E-V+1 (2)

where *M* is the *’meshness’* coefficient; *F* is the number of faces of the planar graph; *E* is the number of edges and *V* is the number of vertices. *M* varies from 0 (tree-like network) to 1 (complete network). A square-like grid network is a compromise between tree-like and complete networks. *M* is usually small because of the lack of triangles or squares in real cities. For example, an average M of 20 real-world cities is 0.219 [33]. As opposed to the ‘meshness’, ‘organic’ indicator *r*_*v*_ is used to measure whether a street network has been planned or not by calculating the ratio of dead ends (*k* = 1) and ‘unfinished’ crossings (*k* = 3). The ‘organic’ *r*_*v*_ is defined as follows [1]:

$$r_{v}=\frac{V\;(1)+V\;(3)}{\sum_{j\neq 2}V\;\left(j\right)}$$ (3)

where *V*(*j*) is the number of vertices of degree *k* = *j*. That is, if this ratio is small, the number of dead ends (*k* = 1) and ‘unfinished’ crossing (*k* = 3) is small compared to the number of regular crossings with *k* = 4 [5, 31]. The vertices with degree *k* = 2 are not counted. Fig 2 shows the histogram of degree distribution for London and Beijing in the primal space, indicating that degree distribution is very peaked around 3–4. To calculate ’*meshness*’ *M* and ’*organic*’ *r*_*v*_ using Eqs (2) and (3). It is found that values of *M* and *r*_*v*_ are approximately 0.26 and 0.63 for Beijing while *M* and *r*_*v*_ for London are 0.22 and 0.89. The investigation result for London is consistent with the study in the literature by [33]. Indicators *M* and *r*_*v*_ suggest the London street network is ‘organic’ as opposed to the lanned street network of Beijing.

### Dual representation and Hierarchical Intersection Continuity Negation (HICN)

To identify roads from street networks in dual space, the well known approaches are *Street Name (SN)* [20] which is based on the symbolic properties of the streets (i.e. street name), and *Intersection Continuity Negotiation (ICN)* [25] which is based on the geometrical properties of the streets (i.e. angle). The merits and shortcomings of the two methods are discussed in [34]. The *SN* method works well on large roads in self-organised cities such as motorways or dual-carriageways but is often misleading in minor roads such as residential roads since it changes several times for the same road. In contrast, the *ICN* method works well on minor roads in planned cities but not on major roads like ring roads. In fact, urban road networks are similar to the cardiovascular network in that they form *hierarchical branching structures* that deliver traffic throughout the city [35]. Accordingly, the research constructs dual graphs for each city using the *Hierarchical Intersection Continuity Negation (HICN)* method [19], which is a hybrid method is developed by combining the geometrical *ICN* with a symbolic *SN* method. The *HICN* first recognises major roads (e.g. ring roads, motorways and dual-carriageway) by applying the *SN* method, then to identify minor roads by employing the *ICN* method. The choice of angular threshold in the study is *π*/2 mainly because the street segments in London and Beijing form angles larger than *π*/2 belonging generally to the same roads. It should be noted that two roads with distinct types should be given different road IDs even though the two roads are aligned. Before creating dual graph via *HICN*, street names in the both datasets is edited manually in order to keep street names consistent in symbolism. That is, one road consisting of a set of continuous segments only shares one name. In the case of London, the *HICN* first is applied to determine motorways (e.g M25) and dual-carriageways (e.g A406 and B515) using the *SN* method. Finally, minor roads is identified by applying the *ICN* method. In the case of the Beijing street network, the *SN* method first is applied to ring roads (from 2th ring to 6th ring), major roads (e.g. primary roads, secondary roads, and tertiary roads), and then the *ICN* method is used to minor roads (e.g. residential roads).

### Betweenness Centrality

**Fig 3:**
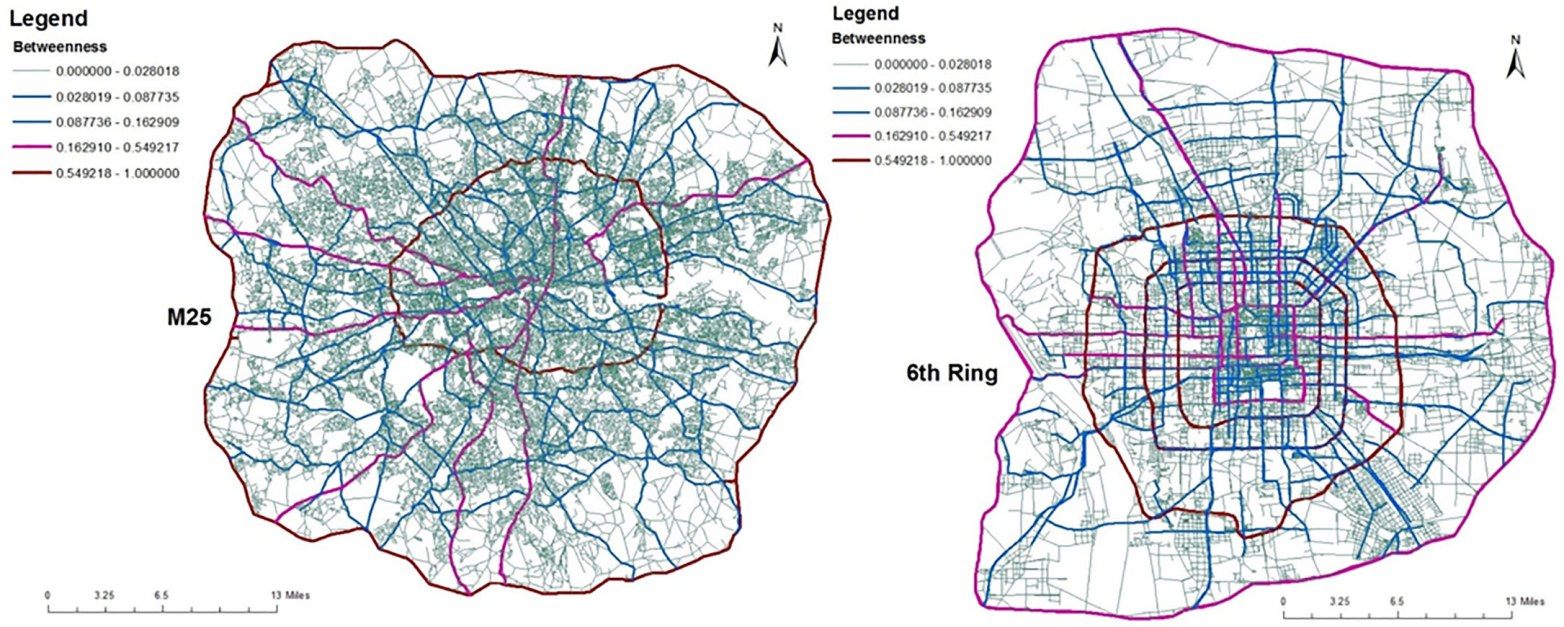
Betweenness centrality on the street networks, which is calculated from the dual space. Left panel: London; Right panel: Beijing. The roads with maximum betweenness centrality in London and Beijing are orbital roads (M25 for London and 4th ring for Beijing). Map composed in ESRI ArcGIS 10.1.

Betweenness centrality is a measure that computes the relative importance of a vertex or edge in a graph in accessibility. The betweenness centrality of a vertex in a graph *G = (V,E)* is defined as:

$$B_{i}=\sum_{j\neq g\in G}\frac{\beta_{jg}\left(i\right)}{\beta_{jg}}$$ (4)

where *B*_*i*_ is the betweenness centrality of a vertex or edge in accessibility; *β*_*jg*_(*i*) is the number of the shortest paths between vertices or edges *j* and *g*, passing through vertex or edge *i*; *β*_*jg*_ is the number of all shortest paths between them. Vertex betweenness centrality in the dual space is calculated and then mapped to the street network in the associated primal space. Keep in mind that a vertex in the dual is equivalent to a road in the primal, which is a collection of edges. Fig 3 shows the maps of betweenness centrality in the dual space for London (left panel) and Beijing (right panel) street networks. Clear hierarchical structure is visible in Fig 3 where in the case of London, the roads with higher betweenness centrality are motorway, and arterials with *radiation* orientation from city centre to periphery and in the case of Beijing the roads with higher betweenness centrality are ring roads and primary roads but they have a *grid-like* shape. Motorway and ring roads connect to arterials and minor roads which connect to lower-capacity surface streets with the design goal of distributing traffic throughout the city.

### Closeness Centrality

**Fig 4:**
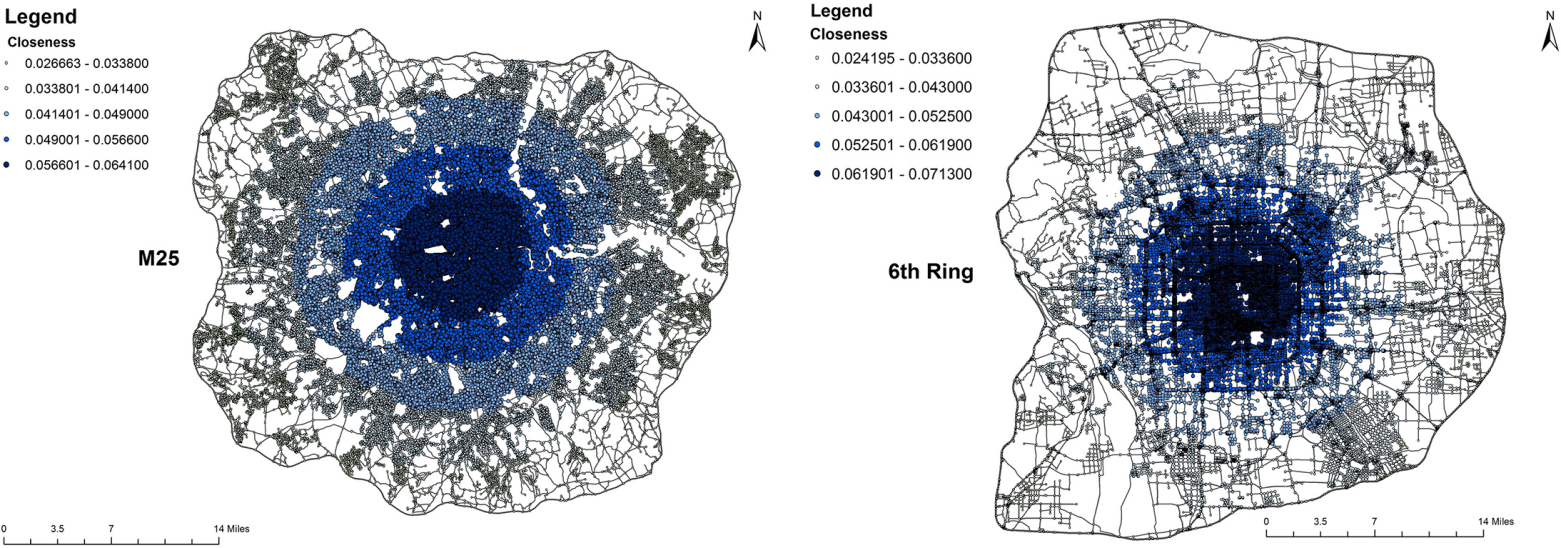
Closeness gradient maps in the primal space. Left panel: London; Right panel: Beijing. Dark dots are places with higher closeness centrality. The colours are decayed from centre to periphery in the both cities. Map composed in ESRI ArcGIS 10.1.

Closeness centrality is a measure of the centrality of a vertices in a network based on the average distance of all shortest paths from that vertex to every other reachable vertex in the network. That is, it measures at the minimum how many steps are needed to access every other vertex from a given vertex. The closeness centrality of a vertex in graph *G = (V,E)* is defined as [36]:

$$C_{i}=V-1/\sum_{i\neq v\in G}d_{iv}$$ (5)

where *d*_*iv*_ is shortest distance in the graph *G* between a given vertex *i* and every other vertex *v*. Vertices that are at a short average length to every other reachable vertex have high closeness centrality. The closeness centrality for isolated vertices is taken to be zero. Fig 4 shows the closeness gradient maps of the London and Beijing, which are calculated from the weighted networks in the primal space. It displays that in London closeness forms the roughly round-like patterns whereas in Beijing closeness forms the square-like patterns along with the periphery of ring roads.

**Fig 5:**
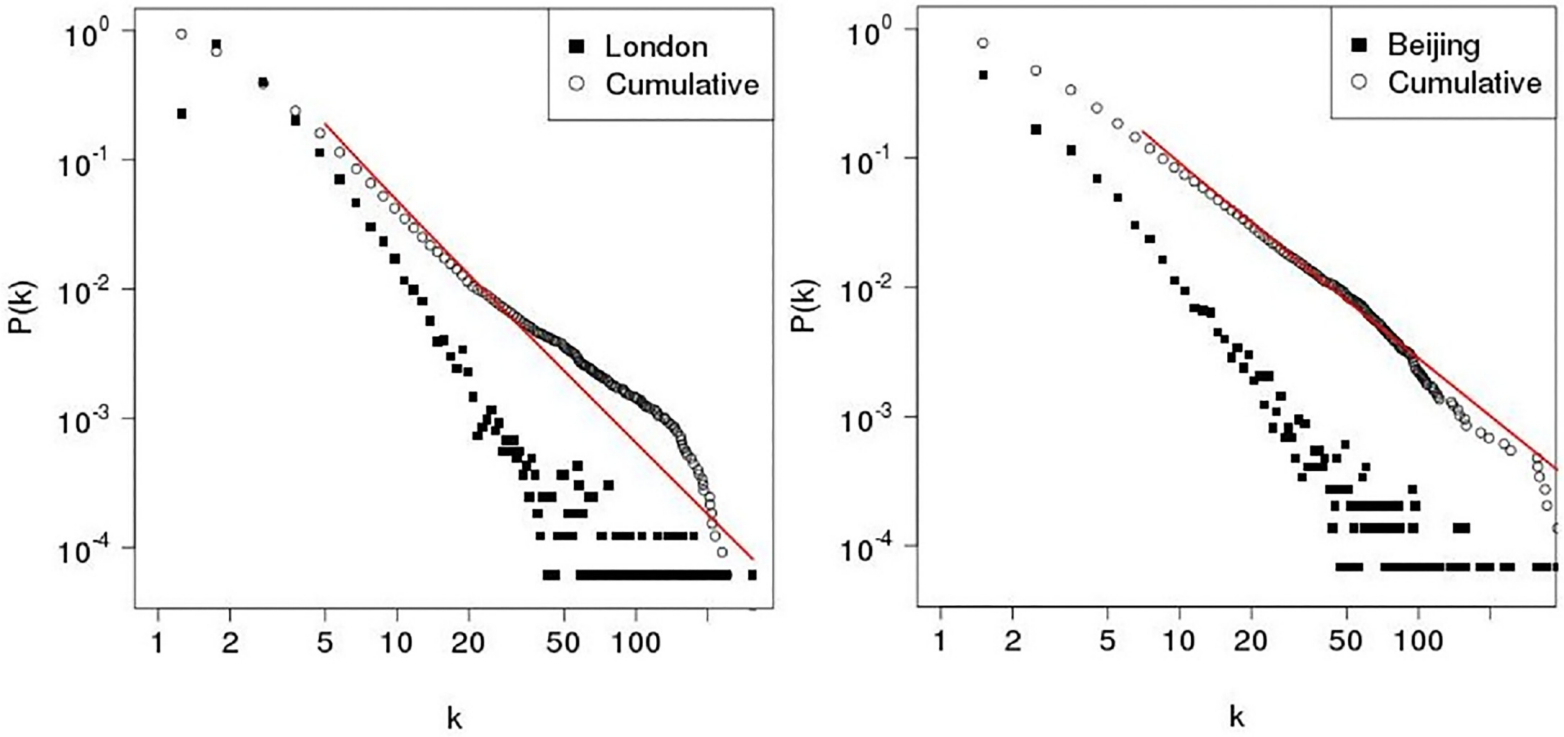
Left panel: Degree density and cumulative distribution in dual space for London. Right panel: Degree density and cumulative distribution in dual space for Beijing.

Using the resulting primal and dual graphs created, the average degrees then are calculated for the two cities. Notably, Beijing shows much higher average degree in the dual space than does London as 〈*k*_*Beijing*_〉 ≈ 5 and 〈*k*_*London*_〉 ≈ 3.6 respectively. Meanwhile, in the primal space the average degrees in London is slightly less than for Beijing, as 〈*k*_*London*_〉 ≈ 2.9 and 〈*k*_*Beijing*_〉 ≈ 3 respectively. The findings are in agreement with the literature [25, 33, 37]. Addtionally, degree connectivity distribution and its corresponding cumulative distribution are calculated for the London and Beijing in the dual space as shown in Fig 5 where there are fat-tailed distributions for the both cities in the dual space where red straight lines are the *theoretical* power law fit. The investigation of how well the fat-tailed distribution can fit power law in comparing with other distributions (e.g. log-normal and exponential) shows that no significant evidence is found for scale-free feature in the dual space. The finding about the degree distribution of the street networks lacking a scale-free property is also in agreement with literature in previous studies [19, 20, 33]. The reason is that the degree connectivity distribution is constrained by the spatial embedding [33].

**Fig 6:**
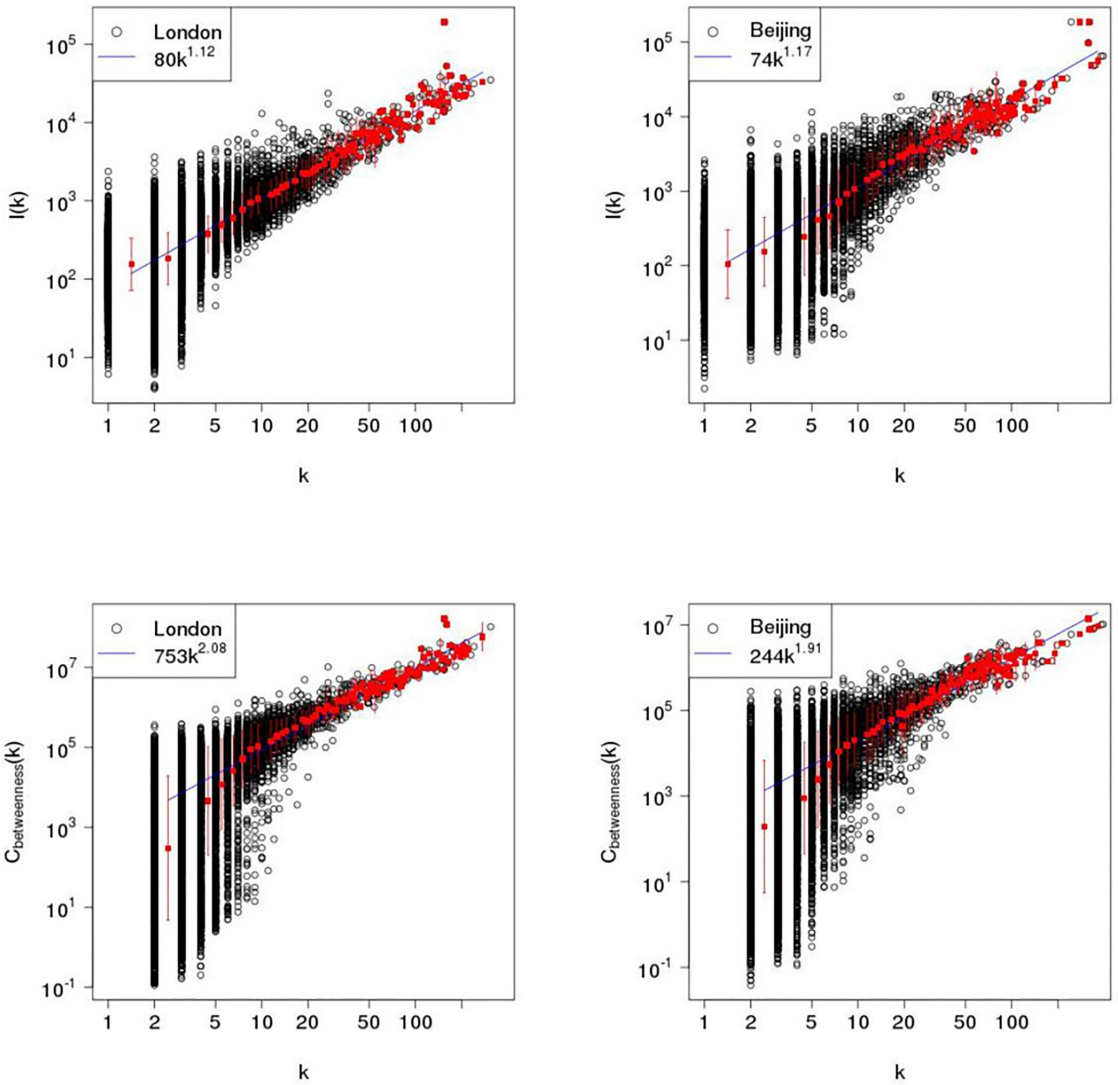
Upper panels: road length of street network as a function of connectivity degree in the dual space for London (Left) and Beijing (Right). Dark point is road length associated with the vertex degree. Red point is value representative of binned data with error bar. Bottom panels: betweenness centrality as a function of connectivity degree in dual space for London (Left) and Beijing (Right). Dark point is betweenness centrality associated with the vertex degree. Red point is value representative of binned data with error bar.

Furthermore, the interconnectedness of graphs of both cities is studied by calculating their *diameters* in the dual space since *diameter* characterises the ability of two vertices to communicate with each other and allows us to see whether the network has small-world property [8]. The diameter in a graph is defined as the longest path between any vertices in a graph. The results show diameter of London is equal to 15, which is smaller than the value of 21 for Beijing, suggesting London has fewer changes in roads across the city than Beijing. Apart from their topological properties, the geometrical properties of both street networks in the primal and dual space also are measured. It appears that London and Beijing are alike in areal extent, with values of 2300 *km* ^2^ and 2200 *km* ^2^ respectively. The total length of roads in London (14752 *km*) is longer than for Beijing (11346 *km*). The spans (network extension in latitude) also are close (51 *km* for London and 54 *km* for Beijing). Complexity of urban street network also can be characterised by fractal geometry [38]. Finally, correlation fractal dimension *D* is calculated based on street intersections, which is defined by [39]

$$D=\lim_{r\to \infty }\frac{log(\sum_{i}p_{i}^{2})}{log(r)}$$ (6)

where the quantity $\sum_{i}\;p_{i}^{2}$ is the probability of finding a pair of points in a cell of length *r*. Correlation fractal dimension in London is 1.888, which is slightly larger than the value of 1.845 for Beijing. Overall, Table 1 gives a summary of topological and geometrical properties of the both street networks using quantitative measures, displaying similarities and distinctions in the primal and dual space. Finally, the relationship between the degree connectivity *k* and road length *l*(*k*) is investigated in the dual space. Remembering that in the dual space a vertex is a collection of edges in the primal space, each of which is associated with a road in the spatial street network. The upper panels of Fig 6 show the road length *l*(*k*) as a function of degree connectivity *k* for London and Beijing, indicating that road length scales with vertex degrees in a superlinear way, with *l*(*m*) ∝ 80 ⋅ *k* ^1.12^ for London and *l*(*m*) ∝ 74 ⋅ *k* ^1.17^ for Beijing. The correlation between the degree connectivity *k* and betweenness centrality *C*(*k*) in the dual space also is studied. The bottom panels of Fig 6 show the betweenness centrality *C*(*k*) as a function of vertex degree *k* in the dual space, revealing a superlinear scaling relationship as well with *C*(*m*) ∝ 753 ⋅ *k* ^2.06^ for London and *C*(*m*) ∝ 244 ⋅ *k* ^1.91^ for Beijing. The superlinear scaling relations in the *dual space* indicate doubling the road connectivity will lead to a larger-than-double increment in road length and betweenness centrality for the both cities.

**Table 1:** Summary of topological and geometrical properties in London and Beijing.

		**Primal**		**Dual**	
	**Indicator**	**London**	**Beijing**	**London**	**Beijing**
Topology	N	74557	44770	35180	15729
	E	107194	67941	62862	38206
	M	0.22	0.26		
	O	0.89	0.63		
	C	1.438	1.518		
	<K>	2.9	3	3.6	5
	K_*max*			309	395
	Diam_top	209	199	15	21
Geometry	A[*km* ^2^]	2300	2200		
	L[*km*]	14752	11346		
	<l>[*m*]	138	167		
	Height[*km*]	51	54		
	Diam_geo [*m*]	67912	73968		
	Fractal_dim	1.888	1.845		

Notation: N = number of nodes, E = number of edges, M = meshness, O = organic, <K> = average degree, C = connectivity, Diam_*top* = topological network diameter, A = network area, L = network length, <l> = average segment length, Height = network extension in latitude, Diam_geo = weighted network diameter, Fractal_Dim = correlation fractal dimension

## Results

Of considerable interest in the study of networked systems is that of their reliability in the face of disruption. To investigate the robustness of the street network, two scenarios—random and intentional attacks are considered to remove road links in the spatial network. The first scenario is to simulate incidents in the transport network by deactivating the edges at random step-by-step 1% in the primal space. That is, edges are picked up in the primal space by a uniform random selection until all the edges in the street networks are erased. The second is to attack roads by eliminating edges in the primal space with high betweenness centrality in the dual space following the method introduced by [26]. Starting from a primal graph with which is associated a dual graph, an edge is removed in the primal graph, which has high betweenness centrality in the dual graph. For each removal, a new primal graph and new dual graph are built for evaluation until betweenness centralities equal to zero in the dual. Notably, in comparison with existing studies [9, 40, 41], a different intentional attack strategy is adopted by introducing a stochastic factor. That is, one of edges in the primal space is chosen for removal using in terms of a certain probability:

$$P\left(i\right)=\frac{B_{i}^{E}}{\sum_{j\;=\;1}^{m}B_{j}^{E}}$$ (7)

where *P*(*i*) is the removal probability of edge *i* in the primal space; $B_{i}^{E}$ is the betweenness centrality of an edge *i*; *m* is the number of edges in the primal space associated to a vertex in the dual space. The primal edge with higher betweenness centrality would be more likely to be selected for removal. For each removal, betweenness centrality in the dual space is updated for next edge removal. In this work, one hundred realisations are carried out for each network and each attack scenario. There is an intrinsically connected questions that naturally arise when one wants to describe quantitatively how a dual space changes when edge is erased in the primal space, which is how to assess the robustness of networks when the edges are eliminated in the primal space. Arguably, if a network is not connected to begin with, it is unlikely to function properly regardless of its intended purpose. By measuring the *Maximum Cluster Size* and *Second Largest Cluster Size* change [14, 15], one can see how fast the network loses its ability of transit when the edges are erased. Meanwhile, efficiency of the network also is calculated to see how well the network performs in the information propagation under the deactivating process.

In order to understand the behaviour of robustness of the two real street networks, two *idealised* models based on the Erdos-Renyi Planar Graph (hereafter ERPG) and the Square Grid (hereafter GRID) are introduced as ERPG can be an idealised approximate for London [21] and GRID can be a reasonable abstract for Beijing [42, 43]. The GRID is defined as *V*_*grid*_ = *N* × *N* vertices, where *N* is the number of vertices on each side, and *E*_*grid*_ = 2*N*(*N* − 1) edges. The average degree of the GRID is deduced from $\langle k_{grid}\rangle =\frac{2E}{V}=\frac{4N(N-1)}{N^{2}}$. The process for building the ERPG and GRID can be found in the literature [21]. In this work, the two networks are set with the following parameters *V*_*erpg*_ = 9,467 and *E*_*erpg*_ = 15,000 for the ERPG, and *V*_*grid*_ = 10,201(*N* = 101) and *E*_*grid*_ = 20,200 for the GRID. In the GRID, the outer boundary of the four sides is considered as one road with the same ID. The Hierarchical Intersection Continuity Negation *HICN* method (see Analysis section) is utilised to identify IDs in the ERPG and GRID as well for creating dual graphs for the ERPG and GRID. The results show that the ERPG and GRID have respectively average degree 〈*k*_*erpg*_〉 ≈ 3.2 and 〈*k*_*grid*_〉 ≈ 3.96 in the primal space and 〈*k*_*erpg*_〉 ≈ 5 and 〈*k*_*grid*_〉 ≈ 100.5 in the dual space. Notably, there is a striking difference in the average degree of the GRID compared with the ERPG in the dual space. Values of *’meshness’* and *’organic’* (see Analysis section) of the ERPG of are approximately 0.29 and 0.52 when compared to 0.49 and 0.04 for the GRID, suggesting the ERPG is more ‘natural’ than the GRID. Furthermore, values of ‘meshness’ and ‘organic’ for the London and Beijing (see Table 1) are compared with those for the ERPG and GRID, showing that meshness values for London and Beijing are lower than for the *idealised* networks. In contrast organic values for the London and Beijing are greater than for the ERPG and GRID. This reflects the fact that structure of the London and Beijing networks is a ‘mixture’ of the ERPG and GRID.

**Fig 7:**
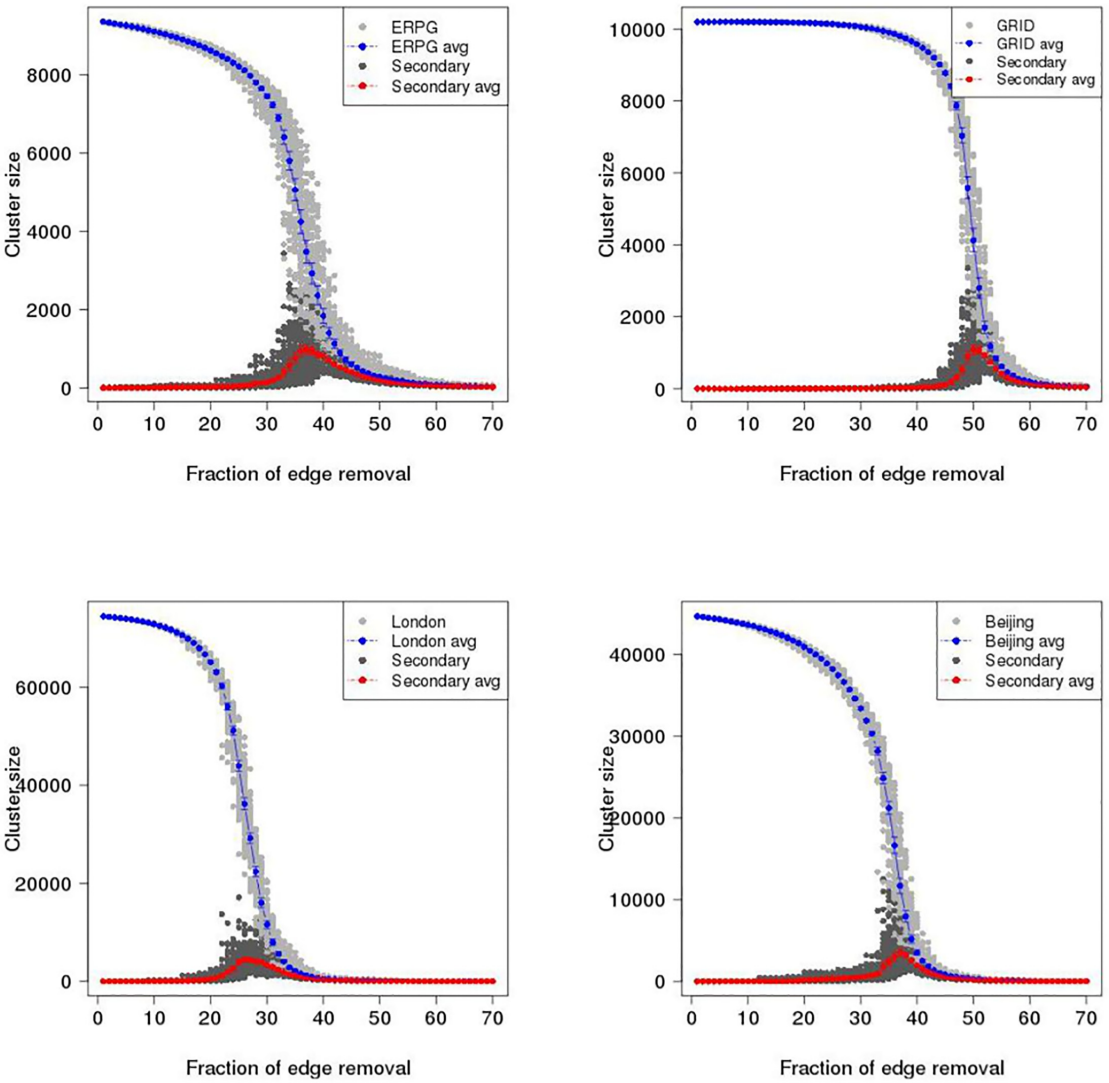
Maximum cluster size and second largest cluster size as a function of proportion of edges removed in the primal space under the random attack scenario. Gray dot is maximum cluster size; Blue dot is maximum cluster size averaged over one hundred realisations with error bar; Dark dot is second largest cluster size; Red dot is second largest cluster size averaged over one hundred realisations with error bar. Upper left panel: ERPG; Upper right panel: GRID; Bottom left panel: London; Bottom right panel: Beijing.

**Fig 8:**
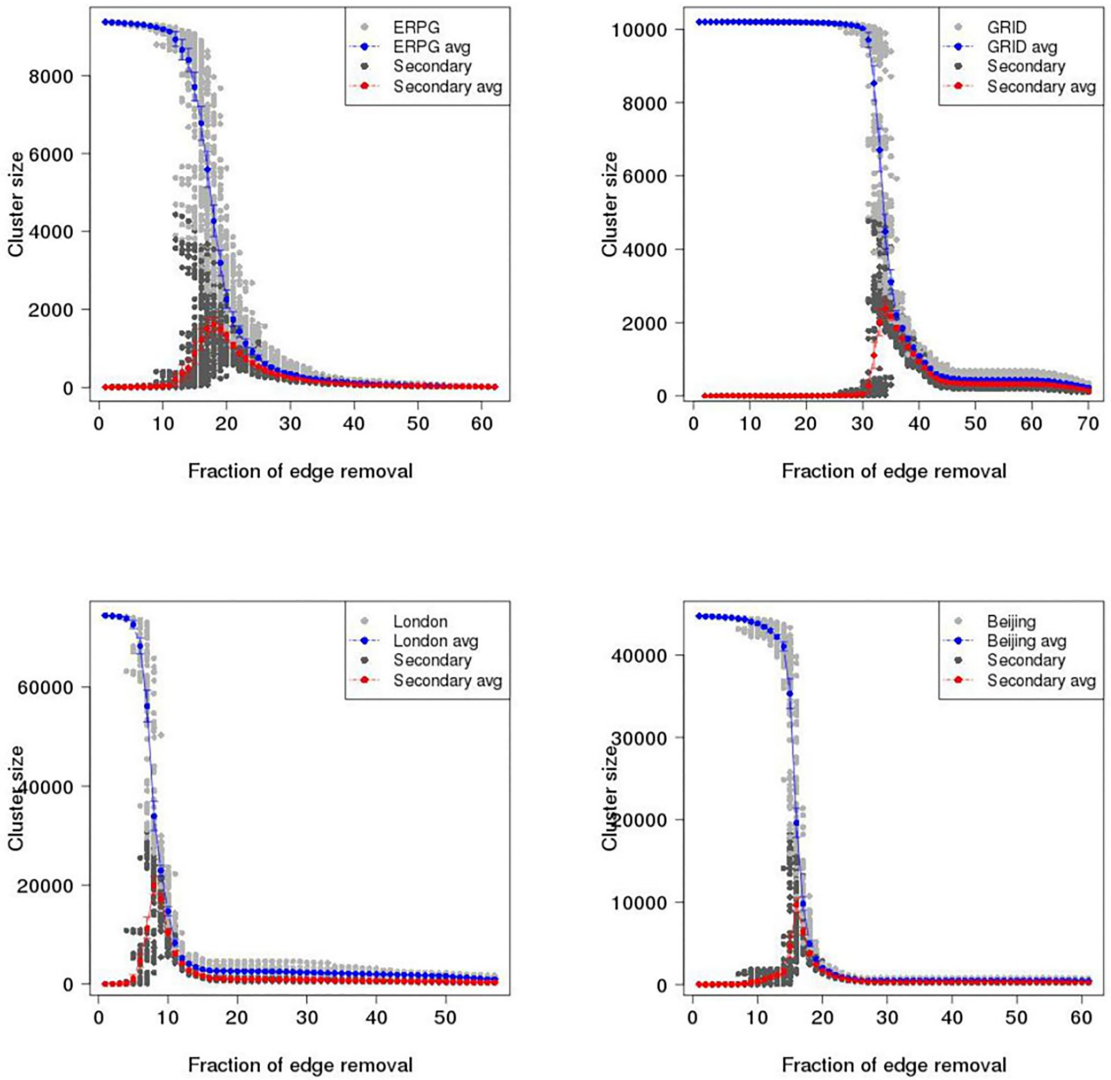
Maximum cluster size and second largest cluster size as a function of proportion of edges removed in the primal space under the intentional attack scenario. Gray dot is maximum cluster size; Blue dot is maximum cluster size averaged over one hundred realisations with error bar; Dark dot is second largest cluster size; Red dot is second largest cluster size averaged over one hundred realisations with error bar. Upper left panel: ERPG; Upper right panel: GRID; Bottom left panel: London; Bottom right panel: Beijing.

**Fig 9:**
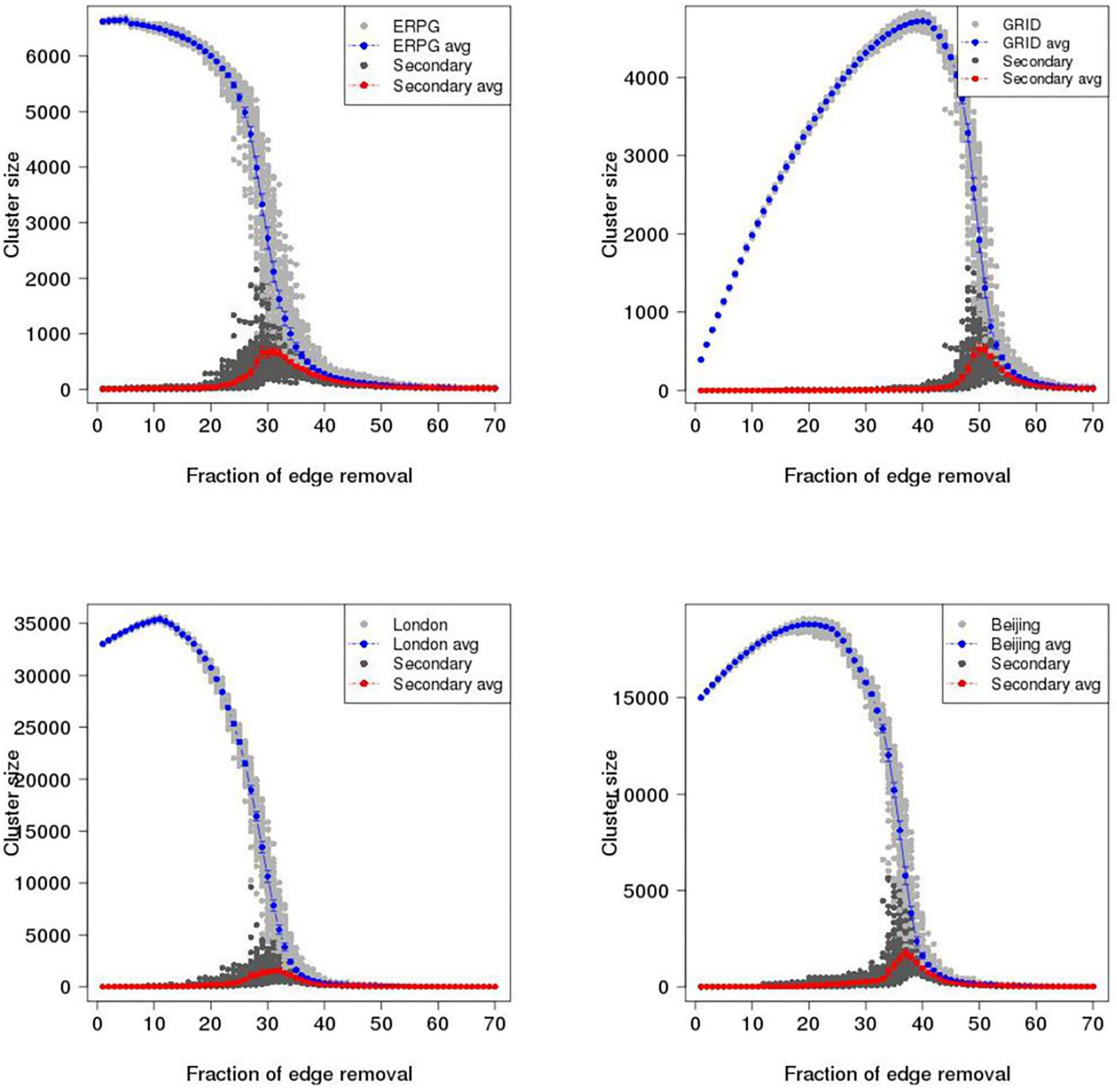
Maximum cluster size and second largest cluster size as a function of proportion of edges removed in the dual space under the random attack scenario. Gray dot is maximum cluster size; Blue dot is averaged maximum cluster size over one hundred realisations with error bar; Dark dot is second largest cluster size; Red dot is averaged second largest cluster size over one hundred realisations with error bar. Upper left panel: ERPG; Upper right panel: GRID; Bottom left panel: London; Bottom right panel: Beijing.

**Fig 10:**
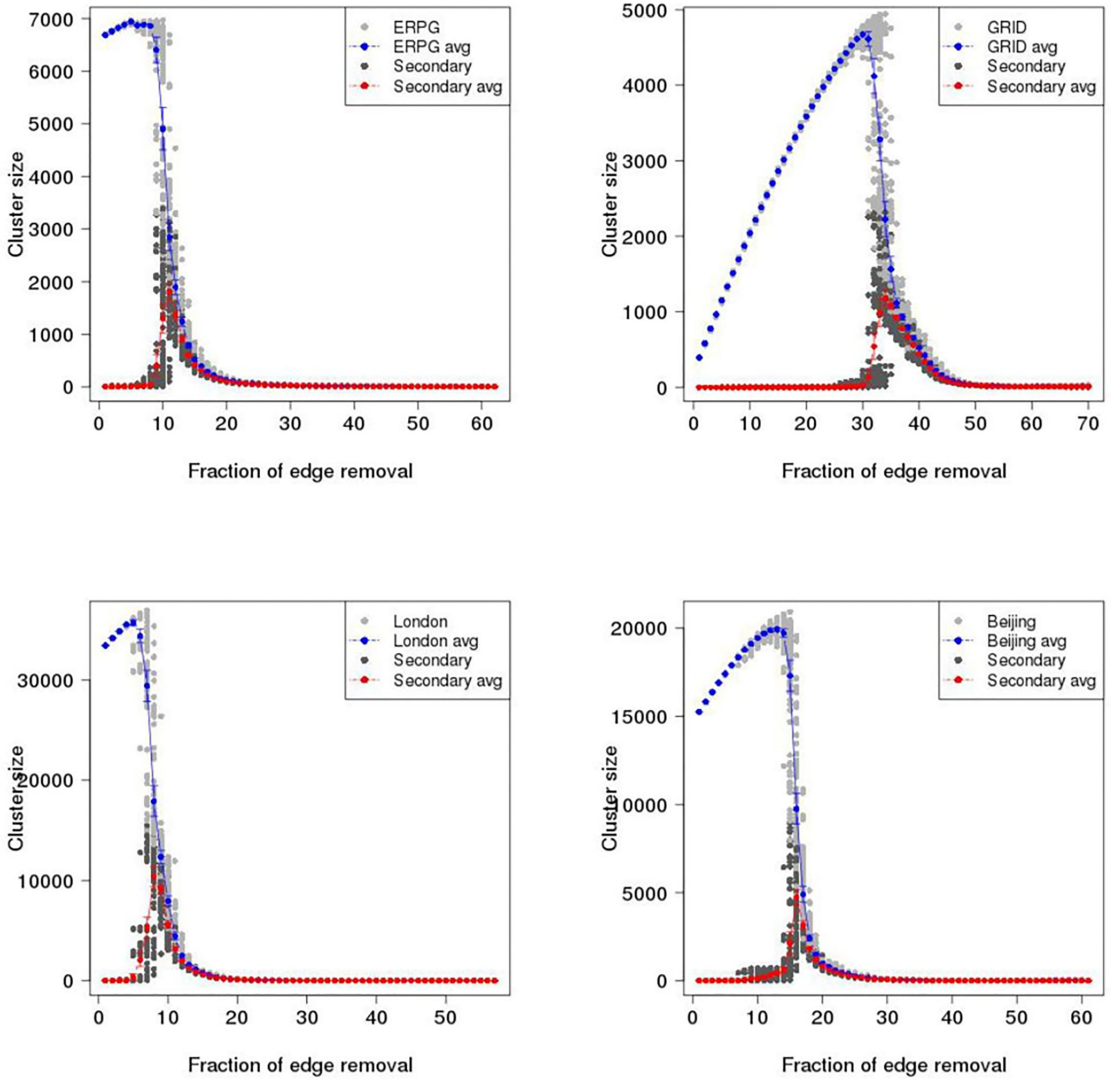
Maximum cluster size and second largest cluster size as a function of proportion of edges removed in the dual space under the intentional attack scenario. Gray dot is maximum cluster size; Blue dot is maximum cluster size averaged over one hundred realisations with error bar; Dark dot is second largest cluster size; Red dot is second largest cluster size averaged over one hundred realisations with error bar. Upper left panel: ERPG; Upper right panel: GRID; Bottom left panel: London; Bottom right panel: Beijing.

**Fig 11:**
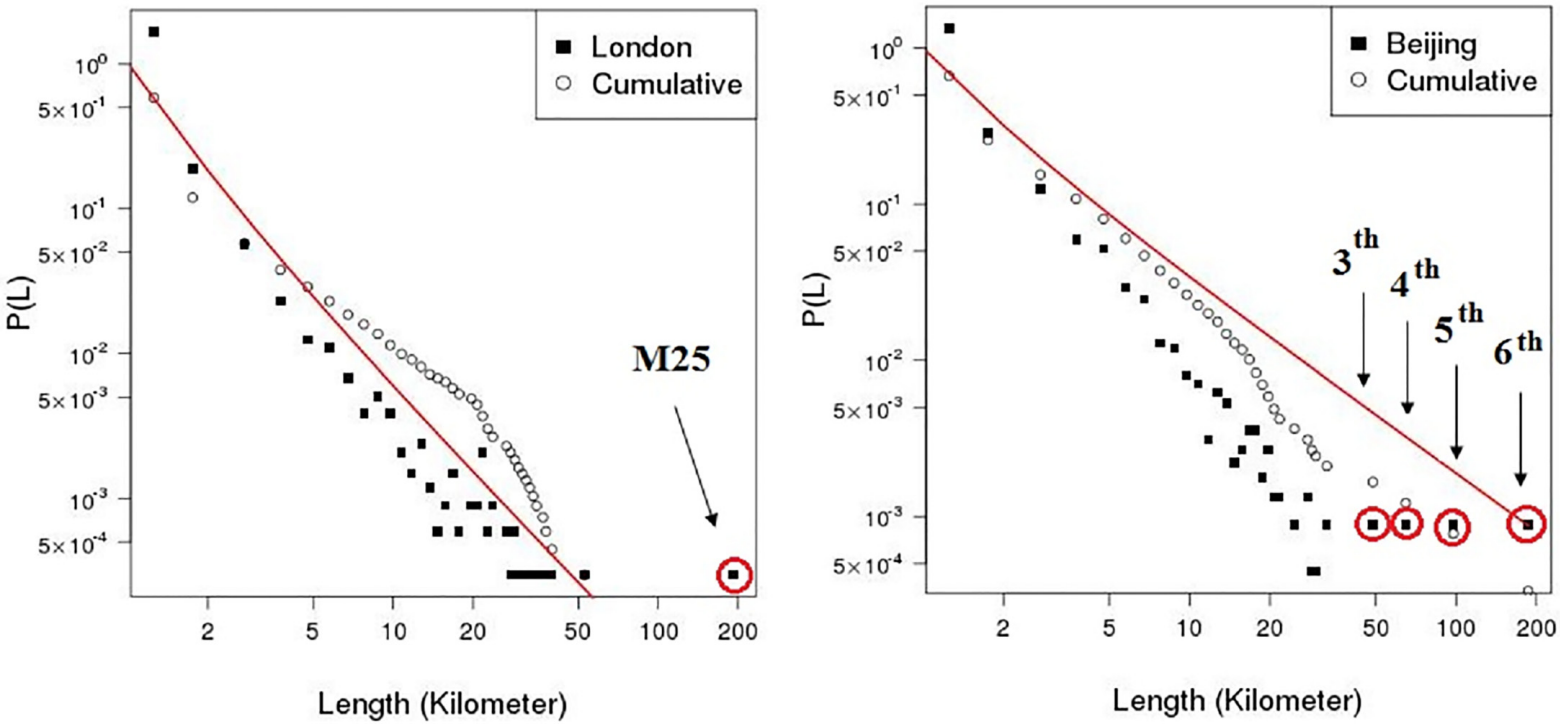
Length distribution in dual space for London and Beijing. Long-range connections across the networks such as M25 in London and 3th–6th rings in Beijing are highlighted by the red circles.

To investigate catastrophic failure in the ERPG, GRID, London and Beijing networks, maximum cluster size (i.e. the number of largest connected vertices in a graph) and second largest cluster size are plotted as a function of proportion of edges removed in the primal space and dual space until 70% of edges are removed. Figs 7 and 8 show the results of random and intended removals with one hundred realisations for the ERPG, GRID, London, and Beijing networks in the primal space under the random attack scenario. A typical behaviour is visible in the Figs 7 and 8 where there is a drop of maximum cluster size accompanied by a peak for the second largest cluster size. It is found that for each realisation the maximum of the secondary component is a critical point where the network breaks in two. After this point the network begins to be fragmented into small pieces and no *spanning cluster* exists. From Fig 7, it can be seen that the GRID performs strongly in the random removal process and then collapses at around 50% of edges removed on average as opposed to 26% in the case of London. Fig 8 shows the results for intended attack removal with one hundred realisations for each network. As I might expect, under the intended attack scenario the networks break down earlier than in the random removal process. Accordingly, the GRID is decomposed at around 34% of edges removed, which is 16% less than in the random removal case shown in the upper left panel of Fig 7. In the case of London, it appears that maximum cluster size drops straight down where the critical point occurs at 8% of edges removed, a reduction of 18% from the random attack scenario. Meanwhile, the breakup of the Beijing and ERPG happens at 16% and 18%, a fall of 21% and 19% respectively. The rates of descent for the Beijing and ERPG case are a bit faster than the London and GRID in the intended removal process. Furthermore, Figs 9 and 10 show the results for the random and intended removals in the dual space. The analysis used the results of one hundred realisations for the ERPG, GRID, London, and Beijing networks. In contrast to the primal space, from Fig 9, it appears that the maximum cluster size in the GRID, London and Beijing does not reduce *monotonically* but increases initially before beginning to decline. The phenomena of ‘increase first and drop after’ in the dual is caused by different definitions to transportation unit (i.e. road) in the primal space and dual space. In the dual space, a vertex corresponds to a collection of edges in the primal space. In the deactivating process, only one edge is picked up for each removal instead of one road. This makes sense particularly on the long-range connections (e.g. M25 in London and 6th ring in Beijing). The breakup of a road will form a ‘new’ road given a new road ID in the dual space. This causes increment of the size of largest component in the dual space until all the edges in the road are erased in the primal space. In fact, the effect of ‘increase first and drop after’ depends mainly on the long-range connections in the dual space. This can be observed in the length distribution in the dual space. Here the length distribution of London and Beijing in the dual space is plotted in Fig 11, which indicates the difference of resilience behaviors in London and Beijing shown in Figs 9 and 10. It is found that long range connections across the networks such as M25 in London and 3th–6th rings in Beijing are strongly skewed towards right (highlighted by the red circles). But, Beijing has more roads with long-range connections than London, causing the difference of resilience behaviors in Figs 9 and 10.

**Fig 12:**
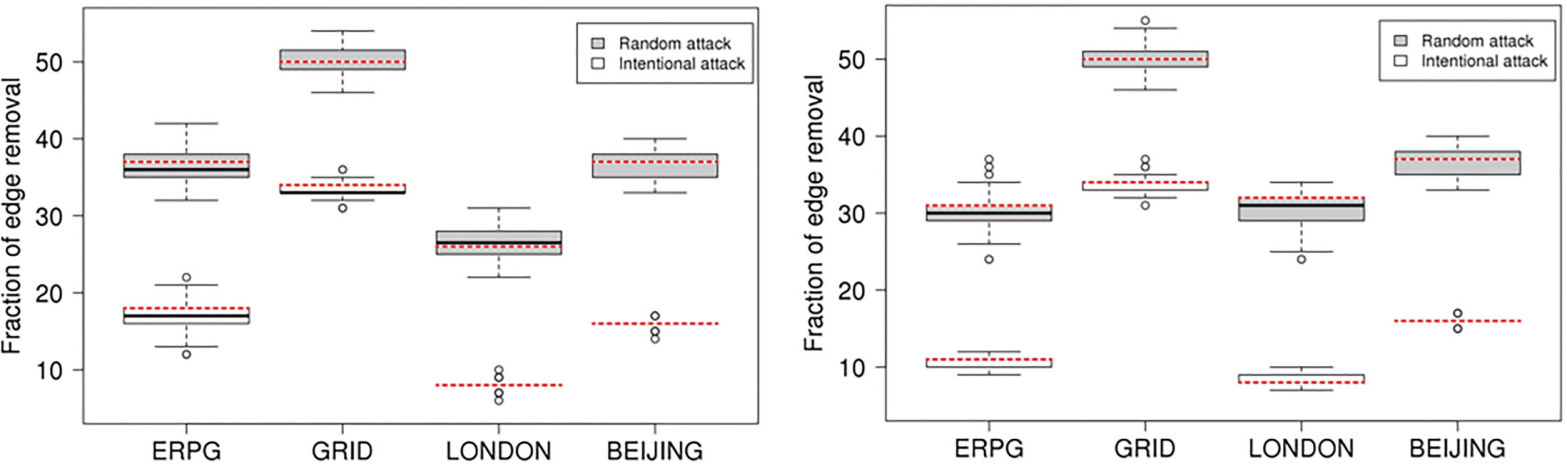
Boxplot of critical points over one hundred realisations under the random and intentional attack scenarios in the primal and dual space for each net. Left panel: primal space. Right panel: dual space. Dashed red lines within the boxes are the critical points calculated by second largest cluster size averaged over one hundred realisations shown in Table A in S1 Appendix.

Strikingly, the GRID has the strongest growth before beginning to decline. The critical point found is at around 50% of removals in the GRID. The second strongest growth is found in the Beijing network with the critical point around 37%. London is in third place with a critical point around 32%, which is similar to the 31% of the ERPG. Accordingly, Beijing is more like the GRID where London is more like the ERPG. It is clear that the GRID and Beijing are more robust than the ERPG and London from the measures of maximum and second largest cluster size. Fig 10 also shows that London and the ERPG look more sensitive than Beijing and the GRID. To investigate critical point variation, Fig 12 shows a boxplot of critical points averaged over one hundred realisations under the random and intended attacks in the both space, where dashed red lines within the boxes are the critical points calculated by the averaged second largest cluster size. Table A in S1 Appendix shows a summary of the critical points averaged over one hundred realisations in the primal and dual space under the random and intended attacks for each network where the differences are observed in the primal and dual space. For critical points of each realisation, it is found that the resilience of different space might be different caused by the distinct definitions to transportation unit (i.e. road) in the primal and dual space, implying that even though the same deactivating process can lead to different resilience effect. In another side, the different resilience of different cities can be explained by the different network properties in the primal and dual space such as spatial structure, average degree, and so on. For example, although London and Beijing have similar degree connectivity distribution (i.e. scale-free like) in the dual space as shown in Fig 5 and small-world feature as shown in Table 1, their resilience behaviour is different. This might be caused by the difference of spatial structure (i.e. tree-like and grid-like structure measured by the indicators of ‘organic’ and ‘meshness’) in the primal space and average degree in the dual space as shown in Table 1. The investigation into the betweenness centrality in the dual space (see Fig 3) also reveals the difference of accessibility structure in London and Beijing.

**Fig 13:**
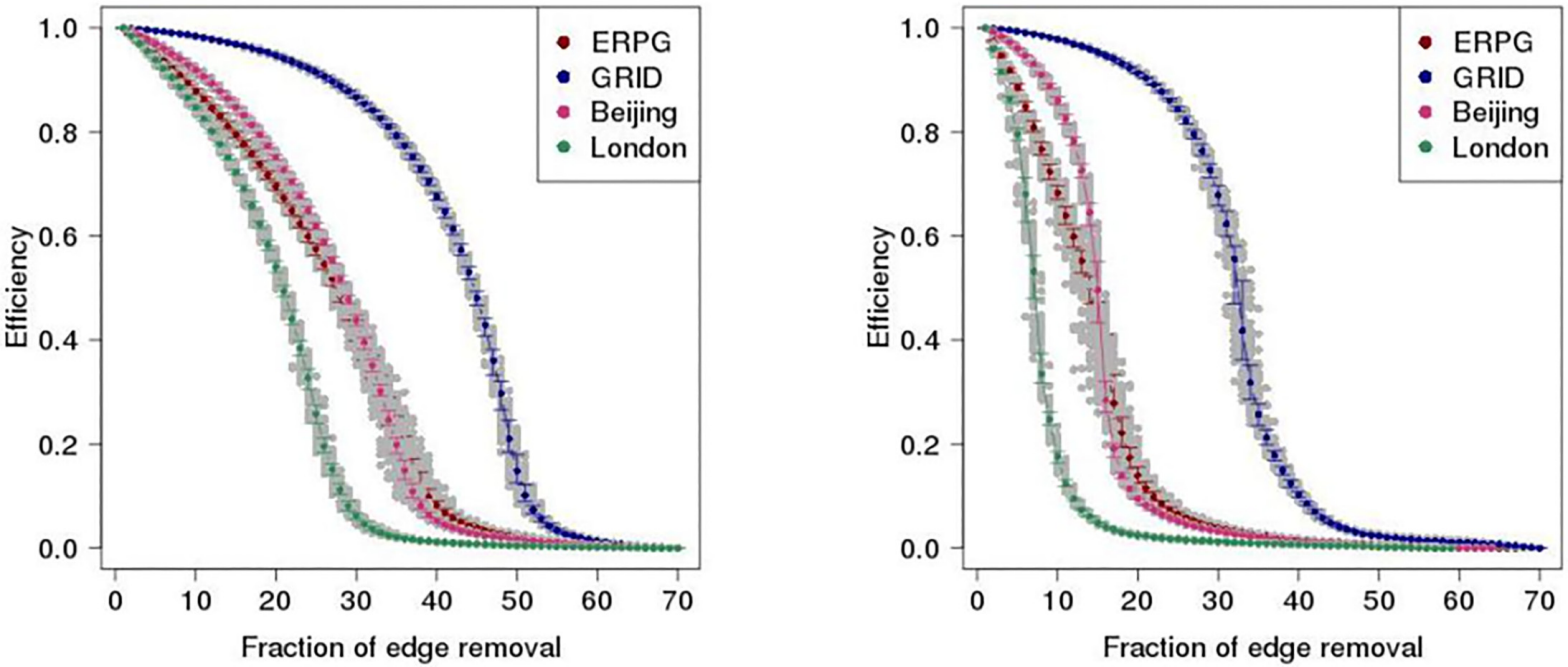
Network efficiency as a function of proportion of edges removed on the street networks under the random (left panel) and intentional (right panel) attack scenarios. Gray dot is network efficiency; Colour dot is network efficiency averaged over one hundred realisations with error bar, where Red is ERPG, Blue is GRID, Pink is Beijing, and Green is London.

Finally, network efficiency is used to assess how well street networks in the primal space perform under the random and intentional attack. Network efficiency is a measure of the information propagation over the entire network, which is based on the assumption that one travels along the shortest routes in a graph *G* [44]. It is easier to transfer information from one vertex to other vertices if they are close to each other. Efficiency of network is defined as the average of sum of inverse of shortest distance in the graph *G = (V,E)* between vertex *i* and *v* [45]:

$$E\left(G\right)=\frac{1}{V(V-1)}\sum_{i\neq v\in G}\frac{1}{d_{iv}}$$ (8)

where *E*(*G*) is global efficiency of network communication. It is normalised to its possible largest values V(V-1) for the fully connected graphs having V(V-1)/2 edges. E(G) is finite even for the disconnected graphs in the removal process. It is expected that the efficiency of network *E*(*G*) declines when an edge is deactivated from the graph *G* in the primal space. Fig 13 shows the efficiency of the network as a function of the proportion of edges removed among one hundred realisations in the London, Beijing, ERPG, and GRID networks, where the gray dot is network efficiency and the coloured dot is network efficiency averaged over one hundred realisations. It can be seen that the Beijing and GRID networks drop more slowly in efficiency than the London and ERPG networks under the both scenarios, suggesting that the former has superior efficiency with respect to network information propagation. The conclusion that top-down planned Beijing is more efficient self-organised London in ability of handling traffic is consistent with the previous studies in the literatures by [42].

## Discussion

The paper has investigated the reliability of the urban street networks via resilience analysis in London and Beijing which are considered as proxies for self-organised and top-down planned cities dominated respectively by natural and central planning urbanisation process. It is found that the reliability of self-organised London is different from the top-down planned Beijing using the measures of maximum and second largest cluster size as shown in Figs 7–10 and network efficiency as shown in Fig 13. The difference of the resilient behaviour of London and Beijing can be explained by the distinctions of network properties in the primal and dual space. For example, although London and Beijing have scale-free like distribution and small world feature in the dual space, spatial structure of London and Beijing in the primal space is different from the measures of ‘organic’ and ‘meshness’ as shown in Table 1. That is, the Beijing street network is dominated by a grid-like structure whereas the London street network tends to a more dendritic and less grid-like structure. In addition, average degree of Beijing in the dual space is much higher than that of London. Spatial pattern of betweenness centrality in the dual space for London also differs from that of Beijing as shown in Fig 3. For example, in London the roads with higher betweenness centrality are motorway, and arterials with *radiation* orientation decaying from city centre to its periphery. However, in Beijing the roads with higher betweenness centrality are ring roads and primary roads with *grid-like* shape. Motorway and ring roads connect to arterials and minor roads which link to lower-capacity surface streets with the design goals of distribution traffic throughout the city. The closeness gradient maps of Fig 4 show the differences of closeness centrality patterns between London and Beijing. For examples, in London the sites with higher closeness centrality locates at the city centre and spread to periphery, forming round-like patterns whereas in Beijing the places with higher closeness centrality lie in the city centre and ring roads, shaping square-like patterns spreading from inner to outskirt.

To investigate whether the reliability of the street network is related to the local connectivity of networks, The property of assortative mixing in the networks is characterised by quantitatively measuring level of assortative mixing. Associative mixing is a pervasive phenomena found in many networks. If high-degree vertices in a network tend to be connected to other high-degree vertices, it shows property of *’assortative mixing’* [46]. Conversely, if high-degree vertices in a network tend to ‘repel’ others high-degree vertices, it is called *’disassortative mixing’*. Degree associative mixing in the ERPG, GRID, London and Beijing is measured, showing that the GRID has highest assortativity 0.659 and the Beijing is 0.184. In contrast, the London and ERPG have assortativity coefficients 0.07 and -0.079 respectively. It is worth noting that networks with higher assortativity also have bigger average degree, which can perform better with respect to its reliability.

Furthermore, the research also studied the correlations between the road connectivity *k*, the road length *l*(*k*) and betweenness centrality *C*(*k*) in the dual space, showing consistent superlinear scaling behaviours for London and Beijing as displayed in Fig 6. The existence of scaling between them is important as it suggests that very general processes are governing the growth of urban street networks no matter it is self-organised London or top-down planned Beijing.

City is a complex system. Its street network, as backbone of city system play a crucial role. The study provides empirical evidence of how shape and appearance of street networks in London and Beijing affects urban transport a from network perspective. The study provides insight into the reliability of self-organised and top-down planned street networks via network analysis. The results can inform those aspects of urban design and planning, where network resilience is of importance.

## Supporting Information

S1 Appendix The supplement contains additional detailed results. (PDF)
