# Supplementary material for: Resilience of Self-Organised and Top-Down Planned Cities—A Case Study on London and Beijing Street Networks
Source: PLoS One. 2015 Dec 18;10(12):e0141736. doi: 10.1371/journal.pone.0141736 (PMC4686176; doi:10.1371/journal.pone.0141736)
Supplement: S1 Appendix — (PDF) [file pone.0141736.s001.pdf]

Resilience of self-organised and top-down planned cities

A case study on London and Beijing street networks

Supplementary Information: S1 Appendix

Jiaqiu Wang[1,*]

[1]Centre for Advanced Spatial Analysis,University College London,W1T 4TJ,London,UK

[*]jiaqiu.wang@ucl.ac.uk

Data Set

The datasets used in the study are *Meridian 2* [1] for London and *OpenStreetMap (OSM)* [2] for Beijing, which are publicly available. The both datasets involve road types and names. For example, in the case of London, roads are classified as A road, B road, Motorway and Minor roads and in the case of Beijing roads are categorised to 31 types such as primary road, secondary road, tertiary road, trunk road, motorway, residential road, and etc. Nevertheless, the roads in the both datasets can be grouped into major roads and minor roads. The street networks in the both datasets first are cleaned by removing links such as footpath, steps, construction, etc for Beijing and duplicated links for London. Then, the roads are fragmented into segments in the both street networks in order to create planar graph. The street names of major roads are edited manually for the both datasets to keep them consistent in symbolism in order to build *dual* representation of a planar graph using *Hierarchical Intersection Continuity Negation (HICN)* method (see the Methods section). That is, a major road in the both datasets consisting of a set of continuous segments shares only one name. Finally, redundant vertices with degree 2 are removed. After that, the London street network is composed by $V = 74557$ vertices and $E = 107194$ edges. And the Beijing street network is composed by $V = 44770$ vertices and $E = 67941$ edges.

Parallel Implementation

For the resilient analysis, it is time-consuming to carry out simulation of hundreds of realisations for each network, particularly on the real size networks like London and Beijing. In this study, the UCL Legion High Performance Computing Facility (Legion@UCL) is used to implement simulation in parallel, enabling the deactivating process

to be performed simultaneously. Table A shows a summary of the critical points averaged over one hundred realisations for each network and each attack scenario in the primal and dual space.

Table A. Critical points (%) averaged over one hundred realisations for each network in the primal and dual space under the random and intentional attack scenarios

	**Primal Space**		**Dual Space**	
	**Random**	**Betweenness**	**Random**	**Betweenness**
ERPG	37	18	31	11
London	26	8	32	8
GRID	50	34	50	34
Beijing	37	16	37	16

The way in which parallelism is exploited depends on the hardware available to the practitioner, but graphics processing units (GPUs) is becoming variable at decreasing cost. Routines optimised for parallel technology that can carry out many of the required computations are readily available. In this study GPU Dijkstra's Single-Source Shortest Path (SSSP) algorithm [3] have been utilised to implement computation of network efficiency on Dell workstation with NVIDIA Quadro 600 graphics card since the runtime of network efficiency increases strongly with network size. The implementation of the GPU SSSP algorithm is available in [4]. Fig 8 in the main text shows a comparison of network efficiencies for the London, Beijing, ERPG, and GRID under the random and intentional attacks.

Network Diameter

Figs A and B show diameter as a function of proportion of edges removed under the random and intentional attack scenarios separately where there is representative behaviour where diameters of networks increase to maximum before declining. For each realisation there is a turning point of diameter *just* before crash where the dual network is broken down into two components. Table B shows the turning points of diameters on average for each network under the random and intentional attack scenarios.

Average Degree

Figs C and D show the average degree of largest clusters as a function of proportion of edges removed under the intentional attacks in the primal space and the dual space. From Fig C, similar patterns are observed between Beijing and the GRID where there are straight declines before becoming flat whereas London and the ERPG looks more alike. However, in the dual space of Fig D it appears that the London and Beijing are more like the ERPG where there is roughly U-bend shape.

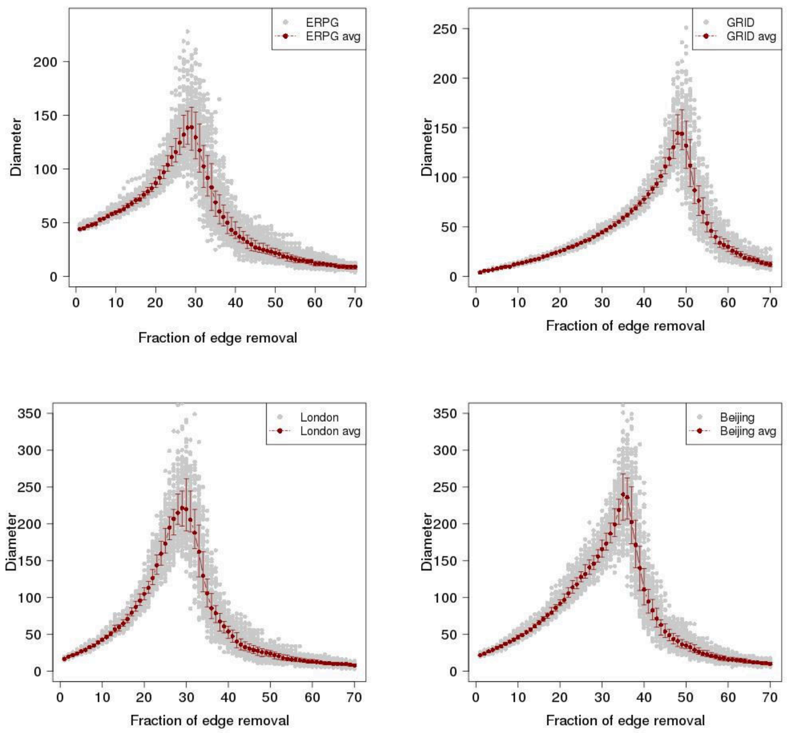

Fig A. Diameter as a function of proportion of edges removed in the dual space under the random attack scenario. Gray dot is diameter; Red dot is diameter averaged over one hundred realisations with error bars. Upper left panel: ERPG; Upper right panel: GRID; Bottom left panel: London; Bottom right panel: Beijing.

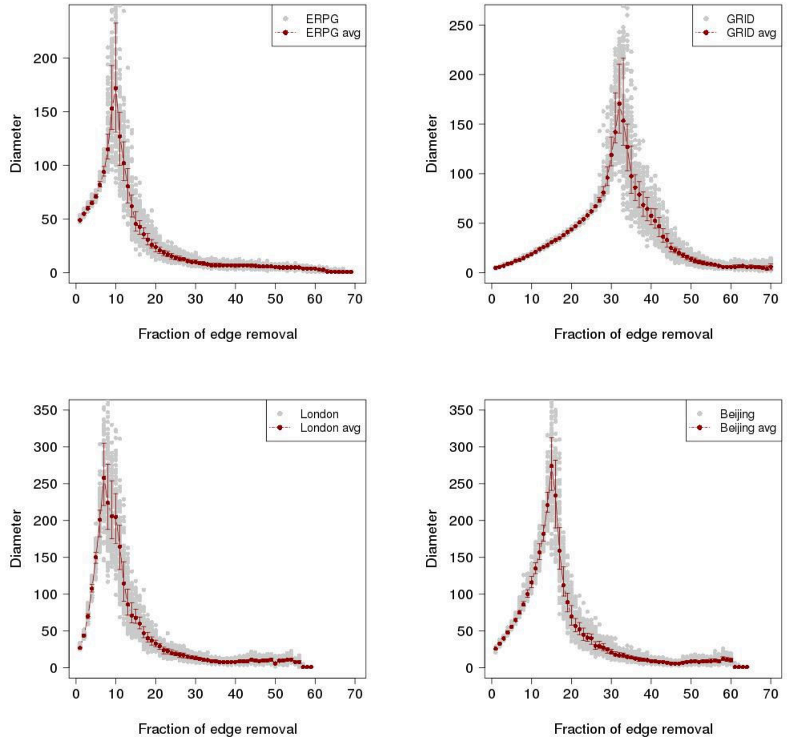

Fig B. Diameter as a function of proportion of edges removed in the dual space under the intentional attack scenario. Gray dot is diameter; Red dot is diameter averaged over one hundred realisations with error bars. Upper left panel: ERPG; Upper right panel: GRID; Bottom left panel: London; Bottom right panel: Beijing.

Table B. Turning points (%) of diameters in the dual space for each network

	Dual Space	
	**Random**	**Betweenness**
ERPG	28	10
London	29	7
GRID	48	32
Beijing	35	15

Betweenness Centrality in ERPG and GRID

Maps of betweenness centrality are reported for the ERPG and GRID, which are calculated from their dual space as shown in Fig E where the roads with highest betweenness centrality (red segments) locate in the centre of the network and the higher-betweenness roads connect to the lower-betweenness roads, showing clear hierarchical structure. However, in the GRID, ring road has highest betweennness and the remaining roads have lower betweenness centrality equally.

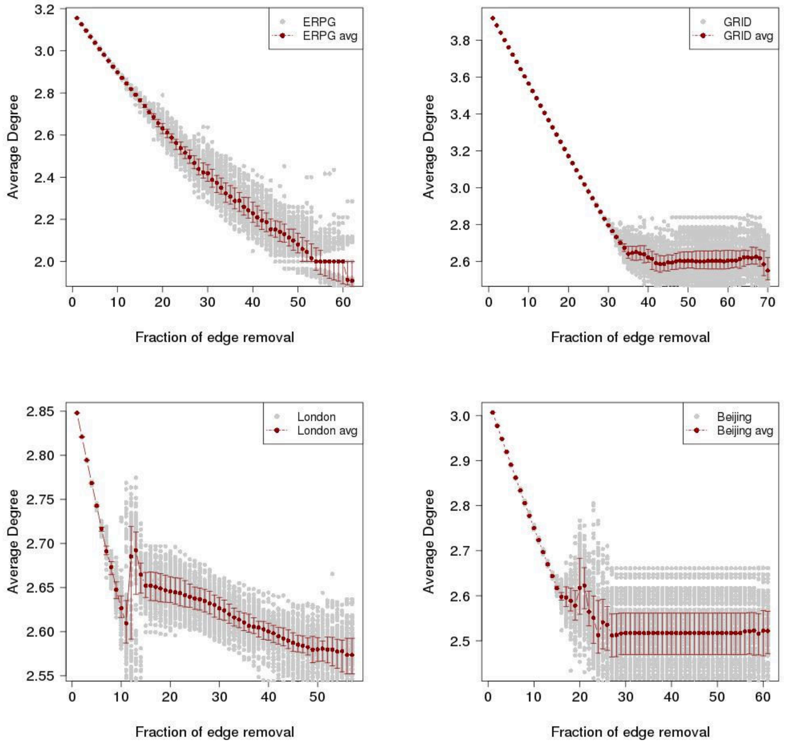

Fig C. Average degree as a function of proportion of edges removed in the primal space under the intentional attack scenario. Gray is diameter; Red is diameter averaged over one hundred realisations with error bars. Upper left panel: ERPG; Upper right panel: GRID; Bottom left panel: London; Bottom right panel: Beijing.

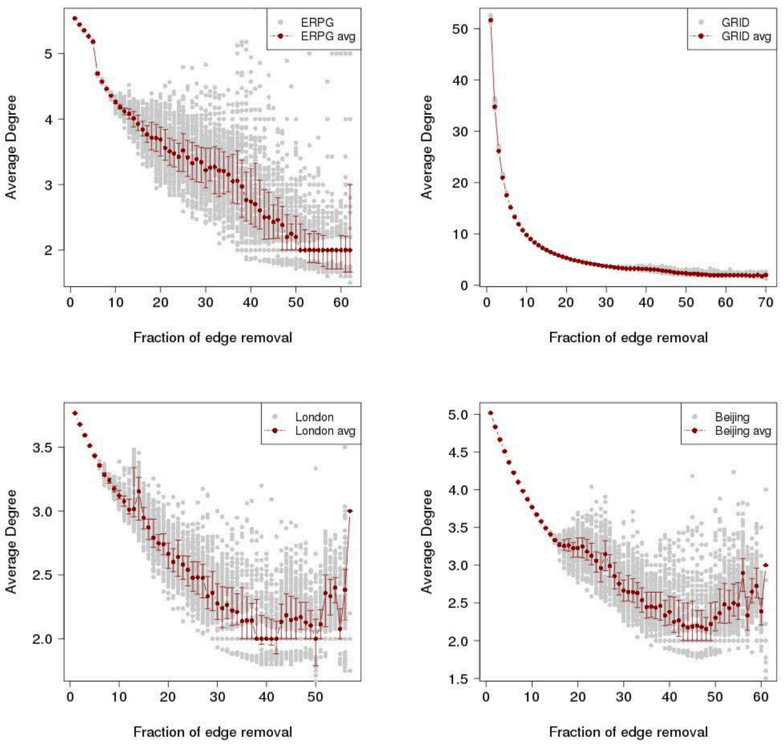

Fig D. Average degree as a function of proportion of edges removed in the dual space under the intentional attack scenario. Gray is diameter; Red is diameter averaged over one hundred realisations with error bars. Upper left panel: ERPG; Upper right panel: GRID; Bottom left panel: London; Bottom right panel: Beijing.

Closeness Centrality in ERPG and GRID

Fig F shows the closeness gradient maps for the ERPG and GRID, which are calculated from the weighted networks where closeness decades from centre to periphery. In the case of GRID, it seems that there are regular rounded patterns whereas in the case of ERPG irregular appearance is observed.

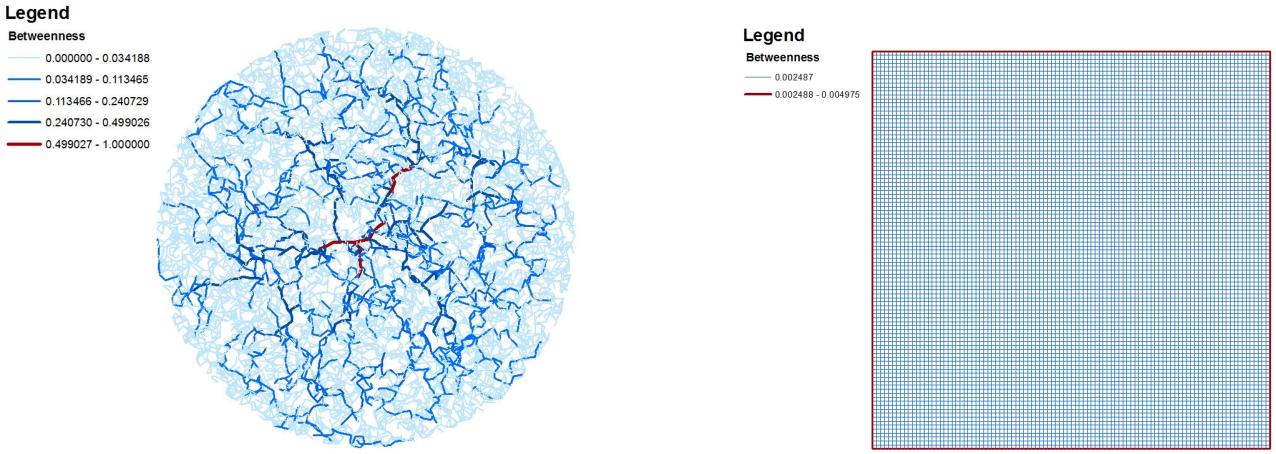

Fig E. Betweenness centrality in the ERPG and GRID, which is calculated from the dual space. Left panel: ERPG; Right panel: GRID.

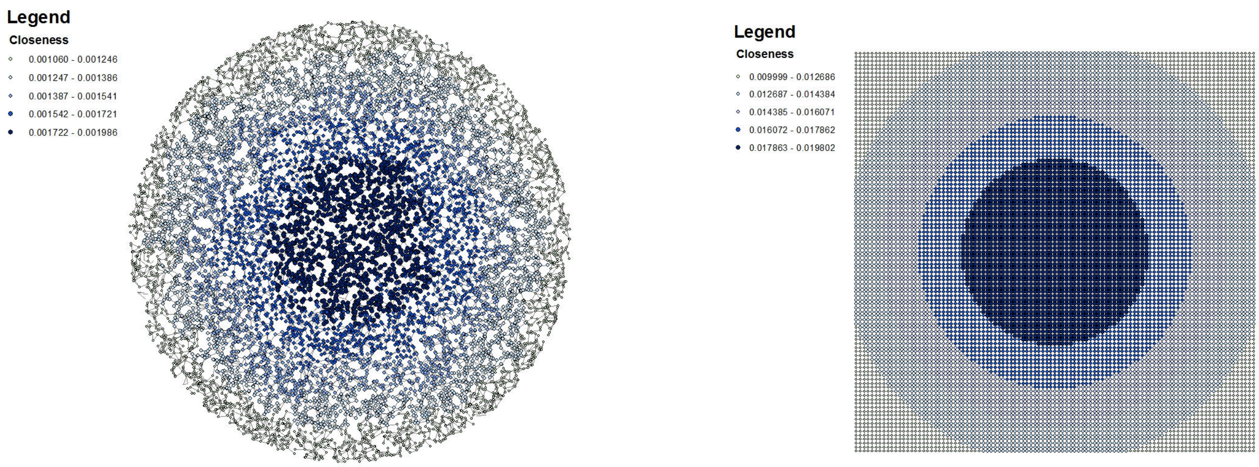

Fig F. Closeness gradient maps in the primal space. Left panel: ERPG; Right panel: GRID. Dark blues are higher-closeness places.

References

[1] URL;. Accessed July, 2013. https://www.ordnancesurvey.co.uk/opendatadownload/products.html.

[2] URL;. Accessed Sep, 2013. http://www.geofabrik.de/.

[3] Harish P, Narayanan P. Accelerating large graph algorithms on the GPU using CUDA. In: High performance computing–HiPC 2007. Springer; 2007. p. 197–208.

[4] URL;. Accessed Jan 2015. http://opencl-book-samples.googlecode.com/svn-history/r17/trunk/src/Chapter_16/Dijkstra/.
